# The mitochondrial genomes of Tortricidae: nucleotide composition, gene variation and phylogenetic performance

**DOI:** 10.1186/s12864-021-08041-y

**Published:** 2021-10-21

**Authors:** Mingsheng Yang, Junhao Li, Silin Su, Hongfei Zhang, Zhengbing Wang, Weili Ding, Lili Li

**Affiliations:** 1 College of Life Science and Agronomy, Zhoukou Normal University, Zhoukou, 466001 Henan China; 2 Finance Office, Zhoukou Normal University, Zhoukou, 466001 Henan China

**Keywords:** Mitogenome, Leaf roller moths, Lepidoptera, Phylogeny

## Abstract

**Background:**

Mitochondrial genomes (mitogenomes) have greatly improved our understanding of the backbone phylogeny of Lepidoptera, but few studies on comparative mitogenomics below the family level have been conducted. Here, we generated 13 mitogenomes of eight tortricid species, reannotated 27 previously reported mitogenomes, and systematically performed a comparative analysis of nucleotide composition, gene variation and phylogenetic performance.

**Results:**

The lengths of completely sequenced mitogenomes ranged from 15,440 bp to 15,778 bp, and the gene content and organization were conserved in Tortricidae and typical for Lepidoptera. Analyses of AT-skew and GC-skew, the effective number of codons and the codon bias index all show a base bias in Tortricidae, with little heterogeneity among the major tortricid groups. Variations in the divergence rates among 13 protein-coding genes of the same tortricid subgroup and of the same PCG among tortricid subgroups were detected. The secondary structures of 22 transfer RNA genes and two ribosomal RNA genes were predicted and comparatively illustrated, showing evolutionary heterogeneity among different RNAs or different regions of the same RNA. The phylogenetic uncertainty of Enarmoniini in Tortricidae was confirmed. The synonymy of Bactrini and Olethreutini was confirmed for the first time, with the representative Bactrini consistently nesting in the Olethreutini clade. *Nad6* exhibits the highest phylogenetic informativeness from the root to the tip of the resulting tree, and the combination of the third coding positions of 13 protein-coding genes shows extremely high phylogenetic informativeness.

**Conclusions:**

This study presents 13 mitogenomes of eight tortricid species and represents the first detailed comparative mitogenomics study of Tortricidae. The results further our understanding of the evolutionary architectures of tortricid mitogenomes and provide a basis for future studies of population genetics and phylogenetic investigations in this group.

**Supplementary Information:**

The online version contains supplementary material available at 10.1186/s12864-021-08041-y.

## Background

The mitochondrial genome (mitogenome) of insects is a circular double-stranded molecule and generally consists of 13 protein-coding genes (PCGs), two ribosomal RNA genes (rRNAs) and 22 transfer RNA genes (tRNAs) [1, 2]. In addition, several noncoding elements, including the control region regulating the replication and transcription of the mitogenome, are present [3]. In recent years, the number of insect mitogenomes sequenced has dramatically increased, greatly improving our understanding of phylogenetics, species delimitation and identification, and population genetics for multiple insect groups [2, 4–8].

In insects, Lepidoptera is the second largest order after Coleoptera, with more than 157,000 described extant species in 43 superfamilies [9–11]. To date, the mitogenomes of approximately 550 lepidopteran species or subspecies have been sequenced and submitted to GenBank (https://www.ncbi.nlm.nih.gov/; last visited in September 2021). Utilizing thisdata, recent efforts of comparative mitogenomics have greatly advanced our understanding of evolution particularly the backbone phylogeny of Lepidoptera [4, 12–16]. However, studies on comparative mitogenomics below the family level are limited in Lepidoptera [2, 17].

The family Tortricidae, the sole member of the superfamily Tortricoidea, consists of approximately 11,365 described extant species, representing one of the most species-rich families in Lepidoptera [9, 18]. Despite the wide acceptance of the three-subfamily classification [19, 20], phylogenetic instabilities exist among prior investigations at the tribe level or below. In contrast to morphology [19–21], for instance, a multilocus study [22] suggests that Bactrini and Endothenini should be synonymized with Olethreutini of Olethreutinae. Using more taxon samplings than Regier et al. [22], Fagua et al. [23] found that the nonmonophyly of Olethreutini still exists. In addition, Tortricidae is notable for containing numerous important pest species that cause large losses in crop and forest production [24, 25], and many pest species (e.g., *Grapholita* spp.) are difficult to distinguish due to their morphological similarity [26]. Thus, in studies addressing species delimitation and the population genetics of these species, molecular markers have been traditionally employed [26–30]. Among these markers, however, only mitochondrial *cox1* and *cox2* are predominately used [26–30], indicating the necessity of screening potential marker candidates through sequencing more tortricid mitogenomes, especially in the context of increasing challenges with the standard *cox1* barcoding marker [31–33].

In Tortricidae, the mitogenomes of only 23 species from five tribes (Archipini, Eucosmini, Grapholitini, Olethreutini and Tortricini) have been sequenced (GenBank, August 2020). Moreover, most studies on tortricid mitogenomes focus on the description of a single mitogenome [34, 35]. Fagua et al. [17] sequenced and annotated six *Choristoneura* species of Archipini and emphasized the mitogenomic divergences of *Choristoneura* spp. associated with habit cooling cycles of the Northern Hemisphere in the Pliocene. Furthermore, the mitogenomic phylogeny based on 19 species from four tortricid tribes shows Eucosmini as paraphyletic.

In this study, we performed a thorough comparative mitogenomic analysis using 13 newly generated mitogenomes of eight tortricid species together with 28 previously reported mitogenomes, of which 27 were reannotated herein where necessary to 1) evaluate the nucleotide composition across major groups of Tortricidae; 2) compare the mitochondrial genetic variation at various taxonomic levels; and 3) assess the phylogenetic performances of various data partitions by constructing the preliminary phylogeny of Tortricidae. The evolutionary architectures of tortricid mitogenomes analysed herein will effectively facilitate further studies on the phylogeny and population genetics of Tortricidae and related groups.

## Results and discussion

### Generation of mitogenome data

Nine complete and four nearly complete mitogenomes were generated and annotated for eight species of Tortricidae. In the nearly complete genomes, we failed to assemble the control regions characterized by highly biased base composition, which was probably due to the disruption of sequencing reactions. All newly generated mitogenomes have been submitted to GenBank with the accession numbers shown in Additional file 1: Table S1.

The 13 mitogenomes each contained 37 typical mitochondrial genes in insects and showed identical gene organization to other reported tortricid mitogenomes which is also typical of Lepidoptera [36]. The lengths of the completely sequenced mitogenomes ranged from 15,440 bp (*L. koenigiana*) to 15,778 bp (*Olethreutes* sp.), compared to other reported tortricid mitogenomes, which have ranged from 15,224 bp (*Lobesia* sp.) to 15,933 bp (*A. fimbriana*). The annotation information of mitogenomes sequenced herein is summarized in Additional file 2: Table S2.

When alignments were conducted among tortricid mitogenomes, some gene boundaries were ambiguous, as previously reported for other mitogenomes. To eliminate their potential impact on subsequent analyses, we carefully checked and revised the annotations of these genes in GenBank mainly according to the methods of Cameron [2]. The reannotation information of 27 previously reported tortricid mitogenomes is summarized in Additional file 3: Table S3.

### Nucleotide composition

**Fig. 1:**
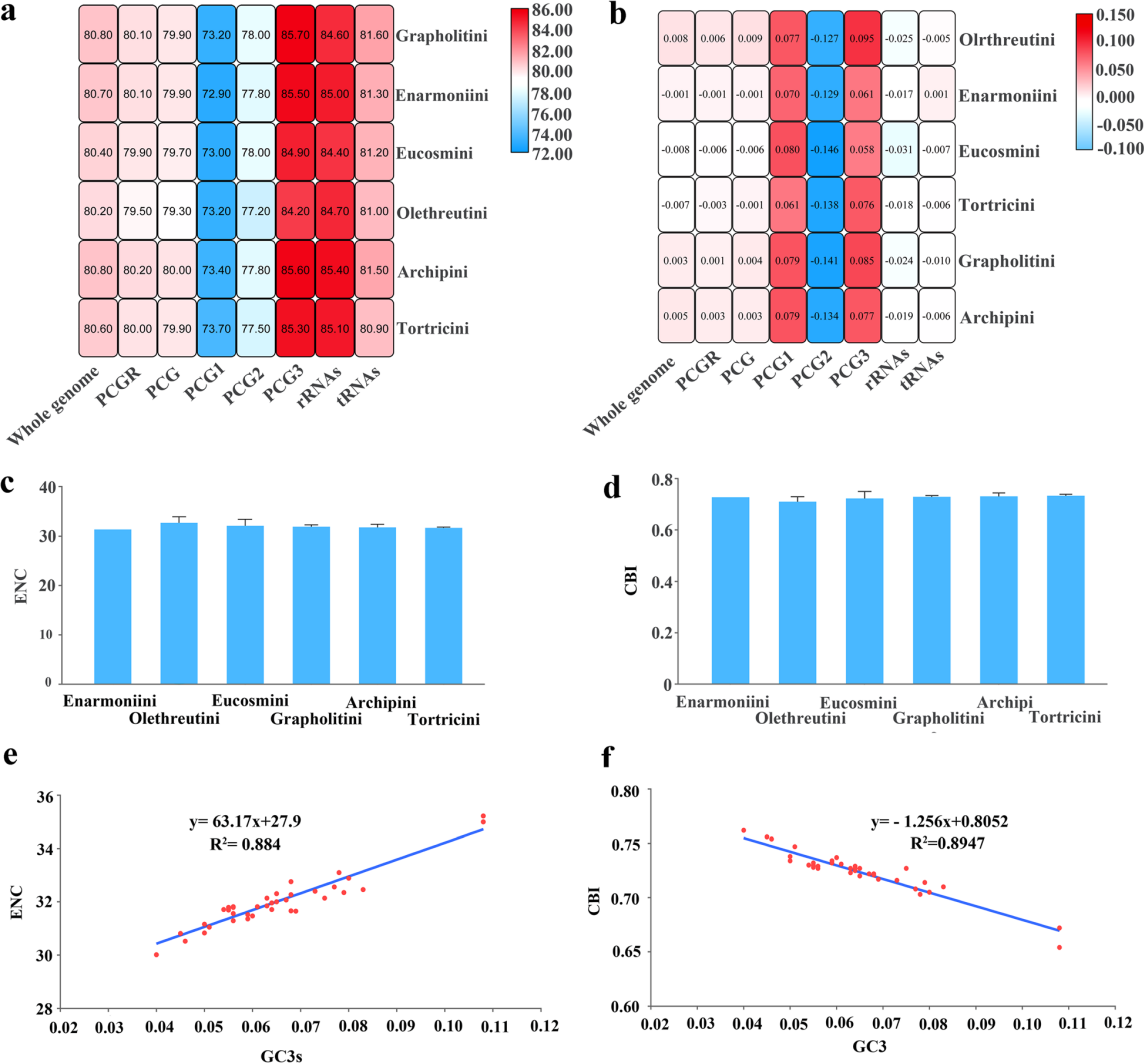
Nucleotide compositions of the tortricid mitogenomes. **a** A + T content; **b** AT-skew; **c** The averaged effective number of codons (ENC) of six tortricid tribes; **d** The averaged codon bias index (CBI) of six tortricid tribes; **e** Scatter plot of the GC content of 3rd codon sites versus ENC; **f** Scatter plot of the GC content of 3rd codon sites versus CBI

The overall nucleotide composition was A (40.5%), G (7.8%), C (11.5%) and T (40.2%), with a highly biased A + T content (80.7%), which is commonly present in insect mitogenomes [3]. Among the six tribes of Tortricidae (Fig. 1a), the A + T contents ranged from 80.2% (Olethreutini) to 80.8% (Archipini and Grapholitini), showing little heterogeneity in nucleotide composition among the tortricid groups analysed in the present study. This is in contrast to some insects from the family to order taxonomic levels [5, 37–40]. Among the three codon positions within the 13 PCGs, the lowest A + T content was found for the first codon position, followed by the second and third codon positions, in accordance with most groups of insects, such as Zygaenoidea of Lepidoptera [14, 15] and Cimicomorpha of Hemiptera [38]. Overall, the rRNAs showed a higher A + T content than the PCGs and tRNAs. The AT-skew and GC-skew are commonly used for evaluating the nucleotide composition of insect mitogenomes [41, 42]. In Tortricidae, negligible AT-skews and negative GC-skews were recognized (Fig. 1b, Additional file 4: Table S4), and six tortricid tribes consistently showed that the second codon positions of 13 PCGs and rRNAs had the lowest values of AT-skew and GC-skew, respectively, a feature commonly present in Lepidoptera [43].

Synonymous codons are generally used with different frequencies [44]. For each amino acid, at least one synonymous codon showed a relative synonymous codon usage (RSCU) value greater than one in tortricid mitogenomes (Additional file 5: Table S5). The RSCU value of UUA encoding Leu, for instance, was higher than the sum of five other synonymous codons, whereas CUC was not used in Enarmoniini. The effective number of codons (ENC) and the codon bias index (CBI) were 30.52–35.22 and 0.65–0.75, respectively, and both exhibited codon usage bias among tortricid mitogenomes to some extent (Fig. 1c, d). Moreover, the positive correlation between the ENC and GC3s and the negative correlation between the CBI and GC3s (Fig. 1e, f) indicate that the genomic G + C content is a significant factor in determining codon bias among organisms [44–46]. In addition, UUA (Leu), AUU (Ile), UUU (Phe), AUA (Met) and AAU (Asn) represented the five most frequently used codons for all tribes, of which a high A + T content obviously contributed to the overall A + T bias of the whole mitogenome.

### Mitochondrial gene variation of Tortricidae

**Fig. 2:**
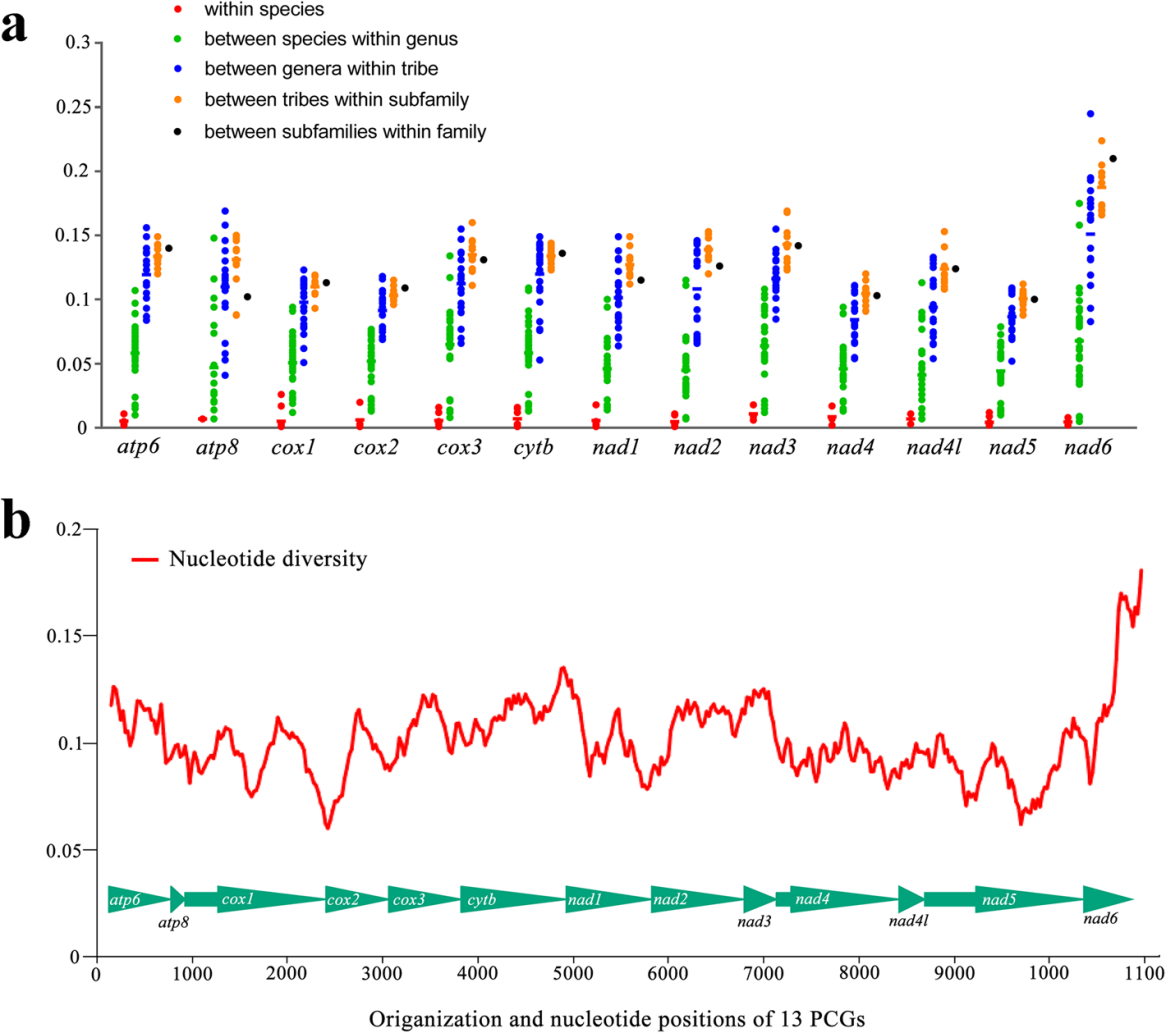
Mitochondrial gene variation of Tortricidae. **a** Mean genetic distances (Kimura-2-parameter) of mitochondrial gene sequences among Tortricidae for different taxonomic ranks; **b** Sliding window analysis shows the value of nucleotide diversity. Gene names and the values of averaged nucleotide diversity are attached

To evaluate the variation patterns of 13 PCGs, genetic distance and nucleotide diversity were calculated. Within species, among species within genera and among genera within tribes, the averaged genetic distances for all PCGs were obviously separated and increased as the taxonomic ranks increased (Fig. 2a). However, the distance gaps were narrower between ranks above the genus level, and *nad1*, *nad2* and *atp8* even showed lower values between subfamilies than between tribes within subfamilies. For instance, the intraspecific distances of 13 PCGs in *G. delineana* ranged from 0 to 0.002. The distances among the three *Archips* species analysed were also variable across 13 PCGs, with the highest value being 0.1 for *nad6*. In contrast, the highest distance of 0.108was for *cox3* between the two *Adoxophyes* species. Our results reflect the variation in rates of divergence among both PCGs and tortricid subgroups, as indicated by Fagua et al. [17] and in other insect groups such as the Miridae of Hemiptera [33].

Regarding the nucleotide diversity of 13 PCGs, sliding window analysis revealed a variable nucleotide diversity both within and among PCGs (Fig. 2b). The average values of nucleotide diversity for individual genes varied from 0.087 (*nad5*) to 0.165 (*nad6*). Similar to *nad5*, *cox2*, *nad4*, *cox1*, *nad4l* and *nad1* showed relatively low nucleotide diversity, whereas the remaining PCGs, as well as *nad6*, exhibited relatively high nucleotide diversity. Nucleotide diversity is commonly used for identifying regions with high nucleotide divergence and could provide guidelines for selecting species-specific markers, especially in taxa with high morphological similarity [47, 48]. The genes with higher levels of divergence identified herein would provide potential marker candidates for population genetics and species delimitation in Tortricidae.

**Fig. 3:**
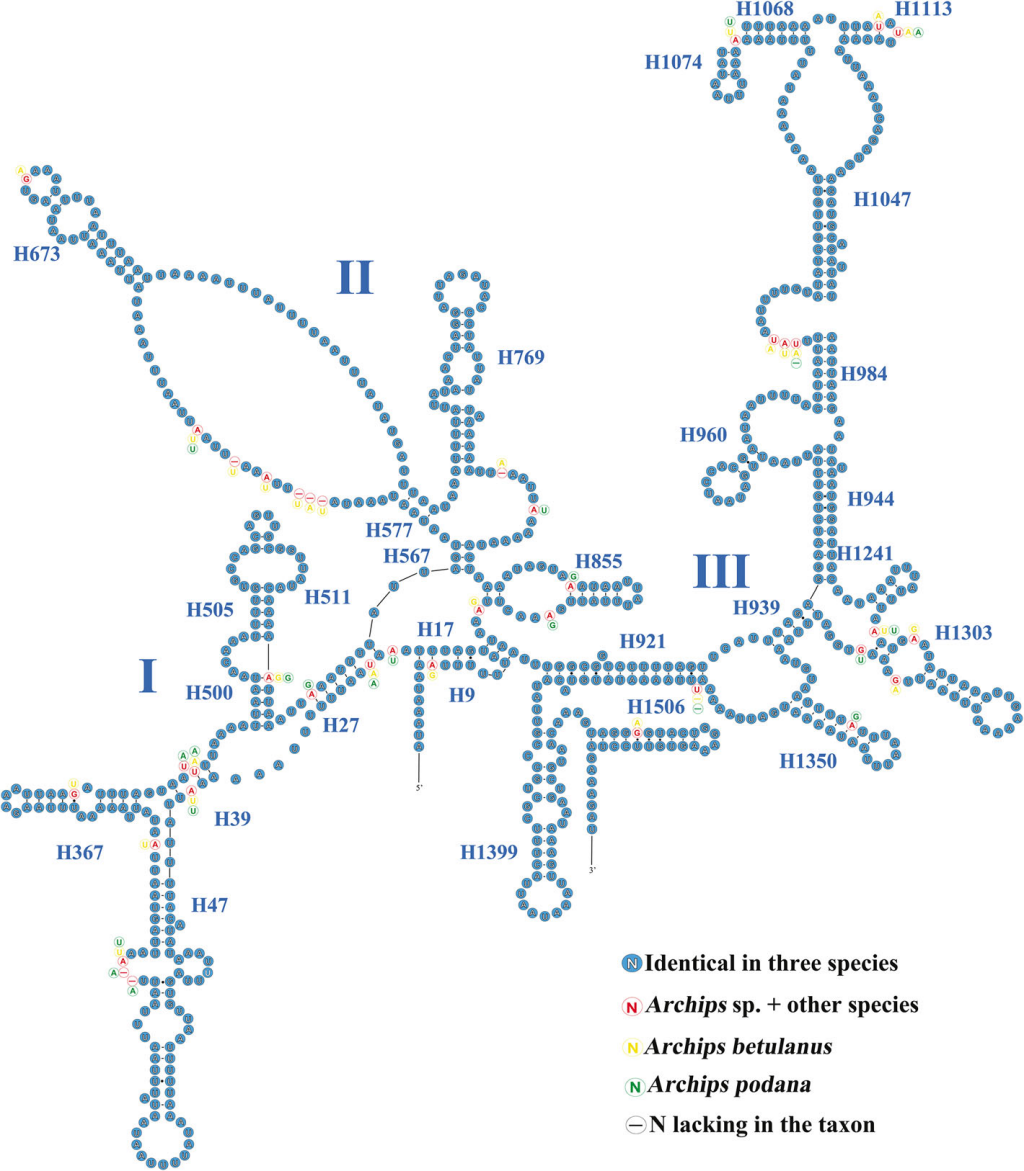
Putative secondary structures of *rrnS* from three *Archips* mitogenomes. Dashes indicate the Watson-Crick base pairs; dots indicate the wobble GU pairs; and the other noncanonical pairs are not marked. Roman numerals denote the conserved domain structure

**Fig. 4:**
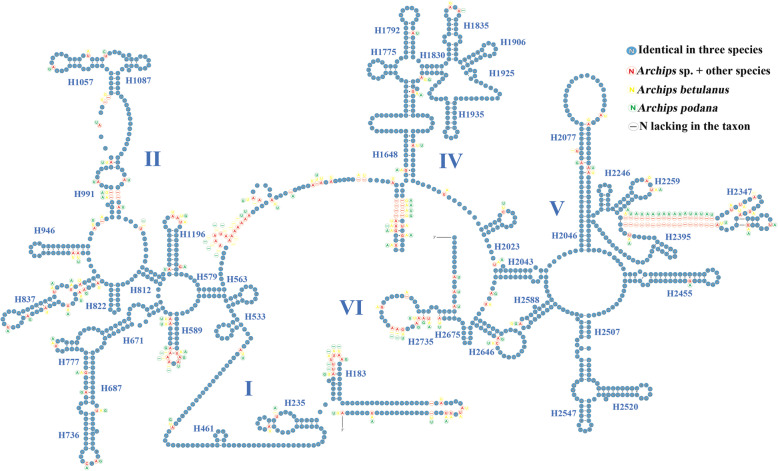
Putative secondary structures of *rrnL* from three *Archips* mitogenomes. Dashes indicate the Watson-Crick base pairs; dots indicate the wobble GU pairs; and the other noncanonical pairs are not marked. Roman numerals denote the conserved domain structure

To characterize the variation distribution of the 24 RNAs, their secondary structures were predicted and comparatively illustrated using three *Archips* species as an example. Comparative tRNA analyses (Additional file 6: Fig. S1) showed that the tRNA structures, including the loss of the DHU arm in *trnS1* (AGN), were highly conserved across the three congeneric mitogenomes, a feature commonly present in lepidopteran insects as well as other metazoans [49, 50]. The nucleotide substitution rates of the 22 tRNAs were different, with *trnW* showing no variation and *trnD* and *trnM* having higher nucleotide substitutions. In addition, for each tRNA, more variable sites were generally present in the TψC arm, TψC loop and DHU loop, a variation distribution similar to the Macroheterocera of Lepidoptera [14, 15]. The secondary structures of the two rRNAswere generally identical to those proposed for some lepidopterans, especially tortricid species [36, 43, 51, 52]. In *rrnS* (Fig. 3), three domains (I–III) were recognized in three *Archips* species (Fig. 4), and the variable sites showed a scatter distribution across three domains. H1047 and H1074 in *rrnS* are highly variable among insect orders [43, 53]. In our analyses, H1074 was highly conserved among the three *Archips* species, indicating that its variation level may increase with higher taxonomic ranks. Regarding the secondary structure of *rrnL* (Fig. 4), five domains (I–II, IV–VI) were detected, with domain III absent, as in other insects [43, 54]. In *rrnL*, a typical feature is the existence of a microsatellite sequence of (TA) n in the stem region of H2347, and the difference in the repeat number among three *Archips* species makes it a highly variable region. This feature has also been found in some *Grapholita* species of Tortricidae [55].

### Phylogenetic informativeness

By adding 13 newly sequenced mitogenomes to 28 existing mitogenomes from GenBank, we performed various phylogenetic analyses to test their phylogenetic implications and to evaluate the phylogenetic performance of different data partitions.

**Fig. 5:**
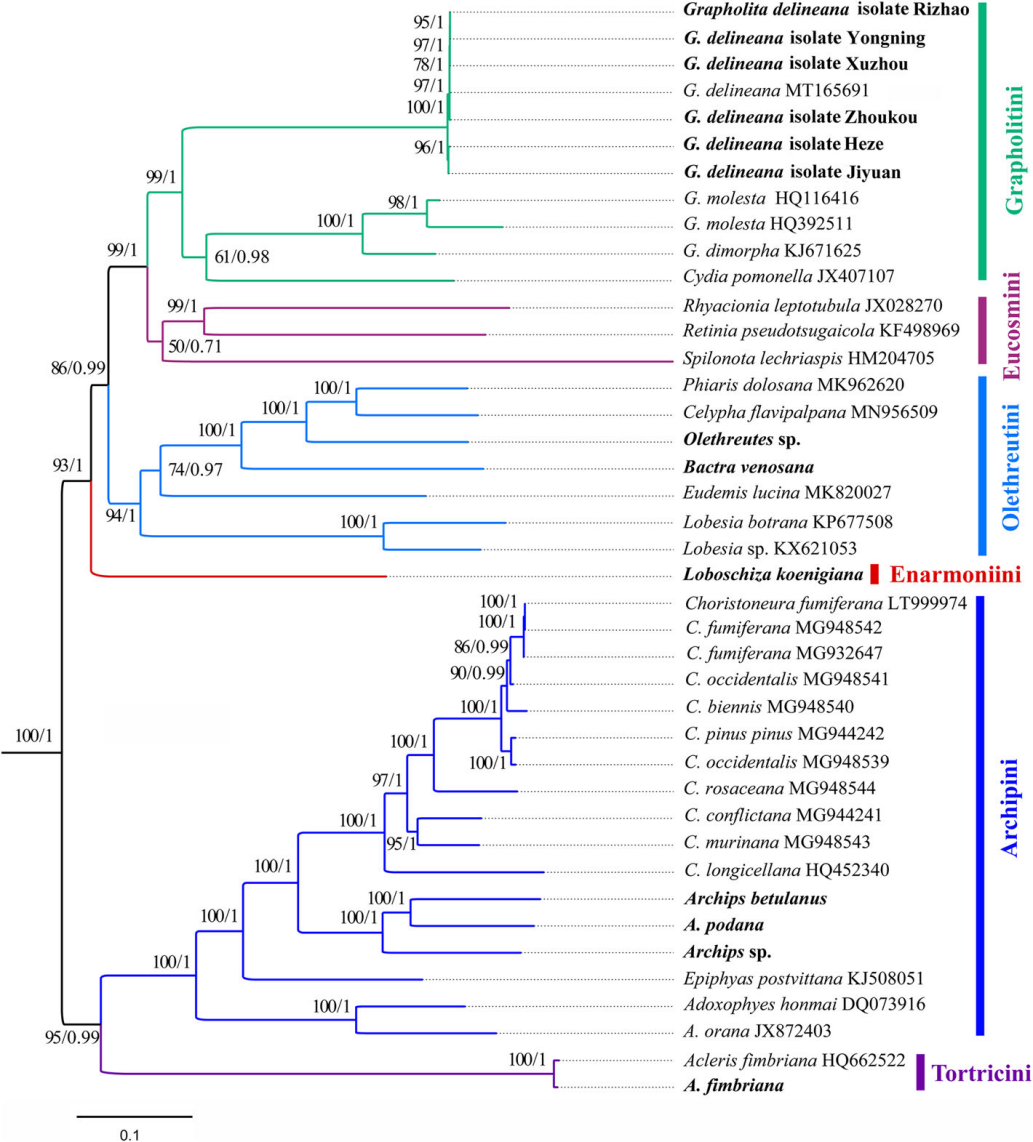
Phylogenetic trees inferred from maximum likelihood and Bayesian inference methods based on the P123RT dataset. Numbers separated by a slash (/) on a node represent the bootstrap support and posterior probability, respectively. The species or individual samples with mitogenomes sequenced in this study are marked in bold

Using four datasets, maximum likelihood (ML) and Bayesian inference (BI) analyses consistently recovered the six tribes and two subfamilies as monophyletic, except for Eucosmini in the ML analysis of the P12 dataset (Fig. 5, Additional file 7–12: Figs. S2–S7). In the subfamily Olethreutinae, four tribes were included in this study, and Enarmoniini was sampled for the first time. (Enarmoniini + (Olethreutini + (Eucosmini + Grapholitini))) was recovered, of which the sister group between Eucosmini and Grapholitini was also supported by Regier et al. [22] and Fagua et al. [23] based on multilocus data. The present study consistently showed *Grapholita* as nonmonophyletic, confirming our recent study [22] and previous multilocus studies [22, 23]. Our results consistently placed *B. venosana*, historically belonging to Bactrini, into Olethreutini, reinforcing the synonymy of the two tribes [22], based on mitogenome evidence for the first time. Six *G. delineana* individuals from different locations were sampled to compare mitochondrial gene variation at the species level, and the phylogenetic results confirmed their position in Grapholitini. Regarding Tortricinae, two tribes were sampled in the present study. In our recent study [55], the position of *Epiphyas* in Archipini was not unstable, being sister to either *Choristoneura* or to *Adoxophyes*. The inclusion of three *Archips* spp. revealed the *Epiphyas* was consistently sister to the (*Choristoneura* + *Archips*) across all datasets, although this relationship remains to be clarified with increased sampling for Archipini. In brief, although the Enarmoniini and Olethreutini were represented for the first time, the present mitogenomic phylogeny only included six of the 19 tribes in Tortricidae [18]. Thus, the relationships among the six tortricid tribes recovered herein definitely need further confirmation with increased sampling.

**Fig. 6:**
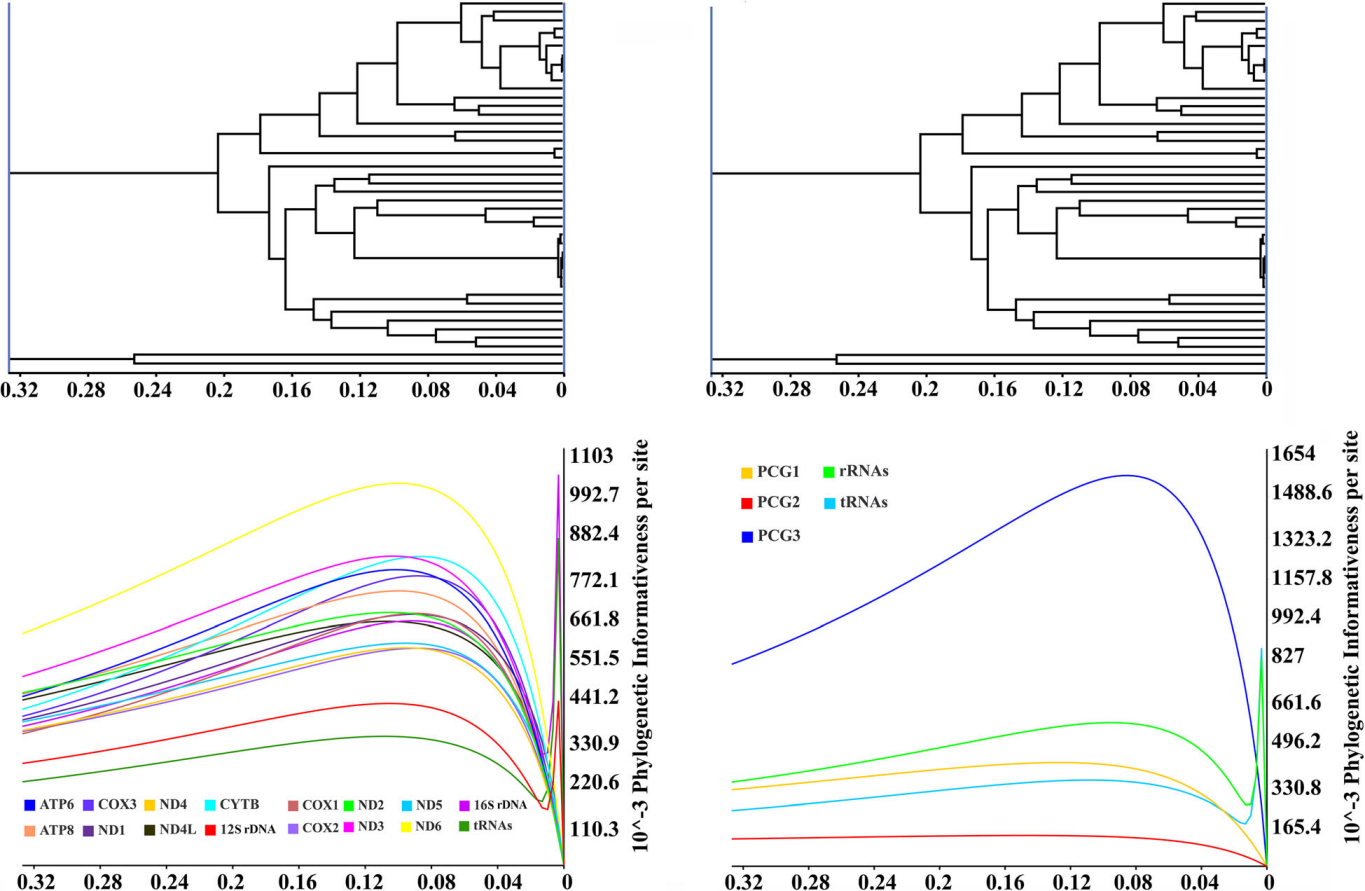
Phylogenetic informative profiles for mitochondrial data partitions. The ultrametric tree was constructed using the P123RT dataset

Despite the high efficiency of using mitogenomes in insect phylogenetic inference, systematic evaluation of the relative contribution of each mitochondrial gene or data partition to the resulting trees has seldom been conducted, especially below the family level. In this study, phylogenetic informativeness (PI) was calculated to assess the contribution of each data partition to the phylogenetic tree from the P123RT dataset (Fig. 6). The PI curves for each data partition were similar in shape, with a steady increase from the root to a peak in the tree showing genus-level relationships and a rapid decrease closer to the tips. Among the 13 PCGs, *nad6* exhibited the highest PI from the root to the tip of the resulting tree, whereas *nad4*, *nad5*, and *cox3* showed lower PI. The high PI of *nad6* was also revealed by Nie et al. [56], who performed a phylogenetic analysis of Galerucinae in Coleoptera. Mitochondrial *rrnL* has been demonstrated to be highly informative in inferring tribe- and subtribe-level relationships in Satyrinae by Yang and Zhang [57, 58]. A similar result was revealed in the present study, and *16S rDNA* had a relatively high PI along with *nad6*. Overall, the 22 tRNAs showed the lowest PI. Furthermore, the three coding positions of 13 PCGs were compared, and the combination of the third coding positions of 13 PCGs showed extremely high PI, as expected. This result was also demonstrated by our phylogenetic analyses which showed higher support values on most nodes of the P123 dataset than on the P12 dataset.

## Methods

### Samples, DNA extraction and mitogenome sequencing

Adults were collected at various locations in China from 2018 to 2019. Specimens were preserved in 100% ethanol in a − 80 °C environment until they were used for DNA extraction. Species identification was conducted through morphology [59, 60] or/and standard mitochondrial *cox1* barcoding [61]. A total of 13 samples representing eight species were selected for sequencing. Among them, *L. koenigiana* is the first sequenced species for Enarmoniini. Three congeneric species, *A. podana*, *A. betulanus* and *Archips* sp. were sampled as an example to evaluate mitochondrial gene variation at the genus level. Six and one samples of *G. delineana* and *A. fimbriana* were included respectively to test the intraspecific variation of mitochondrial genes among population individuals. In addition, two Olethreutini species were selected. *Olethreutes* sp. represented the first sequenced species of Olethreutes, the nominal genus of Olethreutini, and *B. venosana*, historically belonging to Bactrini, was included to test the synonym of Bactrini and Olethreutini proposed by Regier et al. [22].

Total genomic DNA was extracted from head and thorax tissues of a single sample using a DNeasy tissue kit (Qiagen, Germany), following the manufacturer’s instructions. The libraries of 13 samples were individually constructed and sequencing was conducted using an Illumina HiSeq 2500 platform with a 150-bp paired-end strategies. Voucher specimens were deposited in the Biology Laboratory of Zhoukou Normal University, China (Additional file 13: Table S6).

### Mitogenome assembly, annotation and analysis

FastQC (http://www.bioinformatics.babraham.ac.uk/projects/fastqc) was used for quality control of the raw sequences. Clean paired reads were obtained using AdapterRemoval version 2 [62] and SOAPdenovo version. 2.01 [63]. Geneious R11 [64] was used for mitogenome assembly with default settings. Briefly, the “map to reference” strategy was selected to map all cleaned reads to an “anchor” that represents the standard mitochondrial *cox1* barcoding sequence amplified earlier using the general insect primer pair Lco1490 (F) and Hco2198 (R) [65]. After iteration up to 100 times with custom sensitivity, a target sequence with high coverage contigs was generated. Then, MEGA X [66] was used to check the beginning and end of the contig sequence to circularize a complete mitochondrial genome after deleting the overlapping sequence.

The mitogenome sequence was annotated using the MITOS webserver with invertebrate genetic code [67]. MEGA X was used to reconfirm gene boundaries by aligning the new mitogenome with previously reported tortricid mitogenomes available in GenBank. The 22 tRNAs and their secondary structures were reidentified using tRNAScan-SE server version 1.21 [68]. The secondary structures of the two rRNAs were inferred following the models of three other lepidopterans with minor modifications [36, 43, 51].

Nucleotide composition and averaged genetic distances under the Kimura-2-parameter model of 13 PCGs were calculated using MEGA X. Strand asymmetry was calculated according to the formulas: AT-skew = [A – T]/[A + T] and GC-skew = [G – C]/[G + C] [41]. Sliding window analysis (a sliding window of 300 bp and a step size of 25 bp) exhibiting nucleotide diversity for each of 13 PCGs from all samples was performed using DNASP version 6.0 [69]. Additionally, DNASP version 6.0 was used to calculate the ratios of nonsynonymous substitution (Ka) and synonymous substitution (Ks) for PCGs. In addition, the effective number of codons (ENC) and the codon bias index (CBI) were measured using CodonW version 1.4.2 [70].

### Phylogenetic analyses

A total of 41 mitogenomes from 30 tortricid species were used in phylogenetic analyses, including 13 newly sequenced mitogenomes and 28 mitogenomes downloaded from GenBank (Table S1). In addition, two species from Cossoidea served as outgroup taxa. Thirteen PCGs were individually aligned with codon-based mode in the TranslatorX online platform [71]. Two rRNAs and 22 tRNAs were independently aligned with the Q-INS-i algorithm as implemented in the MAFFT online platform [72]. MEGA X was used to check all alignments, and PhyloSuite version 1.2.1 [73] was employed to generate four datasets: 1) P12: the first and second codon positions of PCGs; 2) P123: all codon positions of PCGs; 3) P123R: all codon positions of PCGs plus two RNAs; and 4) P123RT: all codon positions of PCGs plus two RNAs and 22 tRNAs. In addition, independent runs for 13 PCGs and two rRNAs were performed to comparatively evaluate their phylogenetic performance.

Maximum likelihood (ML) analyses were conducted using IQ-TREE 2.0.4 [74] under the partitioning schemes and corresponding substitution models (Additional file 14: Table S7) determined by ModelFinder [75]. Branch supports were calculated using 1000 ultrafast bootstrap replicates [76]. Bayesian inference (BI) analyses were performed with MrBayes version 3.2.6 [77] with the partitioned models (Additional file 15: Table S8) determined by PartitionFinder version 2.1.1 [78]. Twelve processors were used to perform three independent runs each with four chains (three heated and one cold) simultaneously for more than 10,000,000 generations sampled every 100 generations. Convergences were considered to be reached when the estimated sample size (ESS) value was above 200 established by Tracer version 1.7 [79] and the potential scale reduction factor (PSRF) approached 1.0 [77]. The first 25% of samples were discarded as burn-in and the remaining trees were used to calculate posterior probabilities in a 50% majority-rule consensus tree.

### Phylogenetic informativeness

Phylogenetic informativeness (PI) profiles were used to quantify the relative contributions of 13 PCGs and two rRNAs to the resulting tree. The peak of the PI distribution is suggested to predict maximum phylogenetic informativeness for corresponding gene partitioning [80]. To obtain PI profiles, PhyDesign [81, 82] was used with the aligned sequences and an ultrametric tree as input files. The ultrametric tree was constructed using BEAST version 1.7.5 [83].

## Supplementary Information

**Additional file 1: Table S1.** The tortricid samples used in phylogenetic analyses.

**Additional file 2: Table S2.** Annotation and comparison of mitochondrial genome organizations of 13 mitogenomes sequenced in this study.

**Additional file 3: Table S3.** Reannotation and comparison of mitochondrial genome organizations of 27 previous sequenced tortricid species.

**Additional file 4: Table S4.** GC-Skew of the mitogenomes in six tortricid tribes.

**Additional file 5: Table S5.** Codon usage pattern in six tortricid tribes analyzed in this study.

**Additional file 6: Figure S1.** Putative secondary structures of tRNAs from three *Archips* mitogenomes. The tRNAs are labeled with the abbreviations of their corresponding amino acids. The tRNA arms are illustrated as for *trnV*. Dashes indicate the Watson-Crick base pairs; dots indicate the wobble GU pairs; and the other non-canonical pairs are not marked.

**Additional file 7: Figure S2.** Phylogenetic tree inferred from maximum likelihood method based on P12 dataset. Numbers on nodes represent the bootstrap supports.

**Additional file 8: Figure S3.** Phylogenetic tree inferred from Bayesian inference method based on P12 dataset. Numbers on nodes represent the posterior probabilities.

**Additional file 9: Figure S4.** Phylogenetic tree inferred from maximum likelihood method based on P123 dataset. Numbers on nodes represent the bootstrap supports.

**Additional file 10: Figure S5.** Phylogenetic tree inferred from Bayesian inference method based on P123 dataset. Numbers on nodes represent the posterior probabilities.

**Additional file 11: Figure S6.** Phylogenetic tree inferred from maximum likelihood method based on P123R dataset. Numbers on nodes represent the bootstrap supports.

**Additional file 12: Figure S7.** Phylogenetic tree inferred from Bayesian inference method based on P123R dataset. Numbers on nodes represent the posterior probabilities.

**Additional file 13: Table S6.** Information of samples sequenced in this study.

**Additional file 14: Table S7.** The partitioning schemes and corresponding substitution models determined by ModelFinder.

**Additional file 15: Table S8.** The partitioning schemes and corresponding substitution models determined by PartitionFinder.

## Data Availability

The data that support the findings of this study are openly available in GenBank of NCBI at https://www.ncbi.nlm.nih.gov, with the reference numbers MH013482, MW936632–MW936633, MW924656–MW924665.

## Abbreviations

Mitogenome: Mitochondrial genome
*atp6* and *atp8*: ATPase subunits 6 and 8
*cob*: Cytochrome b
*cox1*–*cox3*: Cytochrome c oxidase subunits I-III
*nad1*–*nad6* and *nad4l*: NADH dehydrogenase subunits 1–6 and 4 L
PCG: Protein-coding gene
*rrnL* and *rrnS*: Large and small subunit ribosomal RNA (rRNA)
tRNA: Transfer RNA
RSCU: Relative synonymous codon usage
BI: Bayesian inference
ML: Maximum likelihood
