# Supplementary material for: The mitochondrial genomes of Tortricidae: nucleotide composition, gene variation and phylogenetic performance
Source: BMC Genomics. 2021 Oct 21;22:755. doi: 10.1186/s12864-021-08041-y (PMC8532297; doi:10.1186/s12864-021-08041-y)
Supplement: Supplementary file 1 — Additional file 1: Table S1. The tortricid samples used in phylogenetic analyses. [file 12864_2021_8041_MOESM1_ESM.docx]

**Table S1. The** **tortricid samples used in phylogenetic analyses**

| **Taxon** | **Genome size**  **(bp)** | **GenBank acc.**  **accession no.** |
| --- | --- | --- |
| **Olethreutinae** |  |  |
| **Enarmoniini** |  |  |
| ***Loboschiza koenigiana*** | **15,440** | **MH013482** |
| **Olethreutini** |  |  |
| *Bactra venosana* | **15,588** | **MW924662** |
| *Olethreutes* sp. | **15,778** | **MW936633** |
| *Lobesia botrana* | 15,229 | KP677508 |
| *Lobesia* sp. | 15,224 | KX621053 |
| *Celypha flavipalpana* | 15,498 | MN956509 |
| *Eudemis lucina* | 16,056 | MK820027 |
| *Phiaris dolosana* | 15,562 | MK962620 |
| **Eucosmini** |  |  |
| *Rhyacionia leptotubula* | 15,877 | JX028270 |
| *Spilonota lechriaspis* | 15,368 | HM204705 |
| *Retinia pseudotsugaicola* | 15,282 | KF498969 |
| **Grapholitini** |  |  |
| *Grapholita dimorpha* | 15,813 | KJ671625 |
| *G. molesta* | 15,776 | HQ116416 |
| *G. molesta* | 15,717 | HQ392511 |
| *G. delineana* | 15,560 | MT165691 |
| ***G. delineana* (Heze)** | **15,054** | **MW924657** |
| ***G. delineana* (Jiyuan)** | **15,054** | **MW924659** |
| ***G. delineana* (Zhoukou)** | **15,521** | **MW924660** |
| ***G. delineana* (Rizhao)** | **15,061** | **MW924661** |
| ***G. delineana* (Xuzhou)** | **15,674** | **MW924663** |
| ***G. delineana* (Yongning)** | **15,663** | **MW924664** |
| *Cydia pomonella* | 15,253 | JX407107 |
| **Tortricinae** |  |  |
| **Archipini** |  |  |
| *Adoxophyes orana* | 15,343 | JX872403 |
| *A. honmai* | 15,680 | DQ073916 |
| ***Archips podana*** | **15,461** | **MW924656** |
| ***A. betulanus*** | **16,089** | **MW924658** |
| ***Archips* sp.** | **15,389** | **MW936632** |
| *Choristoneura fumiferana* | 15,304 | MG948542 |
| *C. fumiferana* | 15,541 | MG932647 |
| *C. fumiferana* | 15,320 | LT999974 |
| *C. conflictana* | 15,541 | MG944241 |
| *C. murinana* | 15,540 | MG948543 |
| *C. rosaceana* | 15,544 | MG948544 |
| *C. pinus pinus* | 15,536 | MG944242 |
| *C. occidentalis* | 15,536 | MG948539 |
| *C. occidentalis* | 15,541 | MG948541 |
| *C. biennis* | 15,533 | MG948540 |
| *C. longicellana* | 15,759 | HQ452340 |
| *Epiphyas postvittana* | 15,451 | KJ508051 |
| **Tortricini** |  |  |
| *Acleris fimbriana* | 15,933 | HQ662522 |
| ***A. fimbriana*** | **15,558** | **MW924665** |

Note: The species with mitogenome sequenced in this study is marked in bold.
