# Supplementary material for: The mitochondrial genomes of Tortricidae: nucleotide composition, gene variation and phylogenetic performance
Source: BMC Genomics. 2021 Oct 21;22:755. doi: 10.1186/s12864-021-08041-y (PMC8532297; doi:10.1186/s12864-021-08041-y)
Supplement: Supplementary file 4 — Additional file 4: Table S4. GC-Skew of the mitogenomes in six tortricid tribes. [file 12864_2021_8041_MOESM4_ESM.doc]

**Table S4. GC Skew of the mitogenomes in six tortricid tribes**

| **Group** | **Whole genome** | **PCGR** | **PCG** | **PCG1** | **PCG2** | **PCG3** | **rRNAs** | **tRNAs** |
| --- | --- | --- | --- | --- | --- | --- | --- | --- |
| **Enarmoniini** | -0.1237 | -0.1658 | -0.1782 | -0.0406 | -0.2398 | -0.2394 | -0.3467 | -0.0753 |
| **Olethreutini** | -0.2020 | -0.1902 | -0.2077 | -0.0597 | -0.2719 | -0.2911 | -0.3595 | -0.0737 |
| **Eucosmini** | -0.2188 | -0.2040 | -0.2217 | -0.0855 | -0.2636 | -0.3113 | -0.3590 | -0.0851 |
| **Grapholitini** | -0.1875 | -0.1717 | -0.1940 | -0.0486 | -0.2603 | -0.2448 | -0.3464 | -0.0652 |
| **Archipini** | -0.1821 | -0.1717 | -0.1900 | -0.0451 | -0.2523 | -0.2639 | -0.3333 | -0.0703 |
| **Tortricini** | -0.1710 | -0.1600 | -0.1741 | -0.0382 | -0.2321 | -0.2381 | -0.3378 | -0.0681 |
