# Supplementary figures and images for: The mitochondrial genomes of Tortricidae: nucleotide composition, gene variation and phylogenetic performance
Source: BMC Genomics. 2021 Oct 21;22:755. doi: 10.1186/s12864-021-08041-y (PMC8532297; doi:10.1186/s12864-021-08041-y)

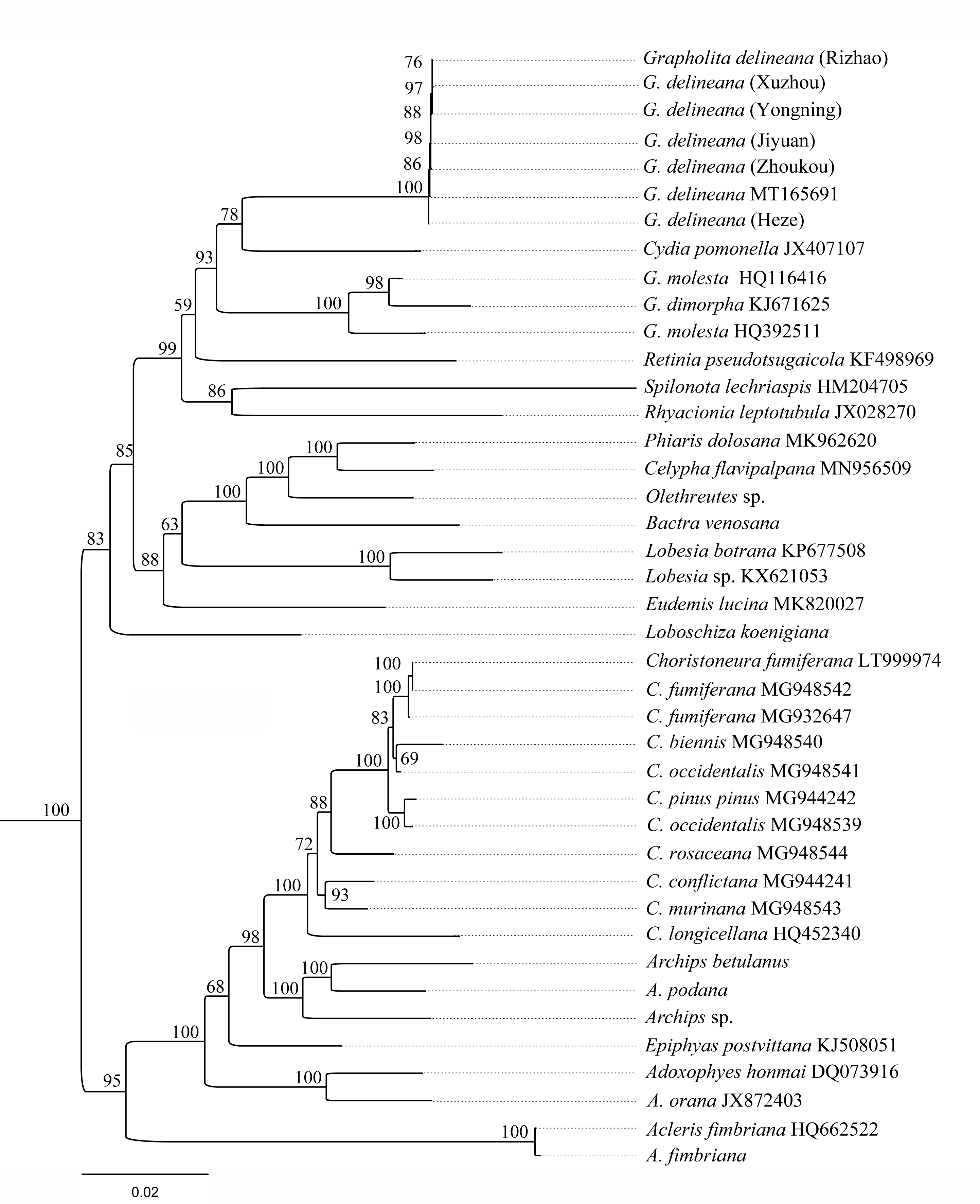

Supplement: Supplementary file 7 — Additional file 7: Figure S2. Phylogenetic tree inferred from maximum likelihood method based on P12 dataset. Numbers on nodes represent the bootstrap supports. [file 12864_2021_8041_MOESM7_ESM.tif]

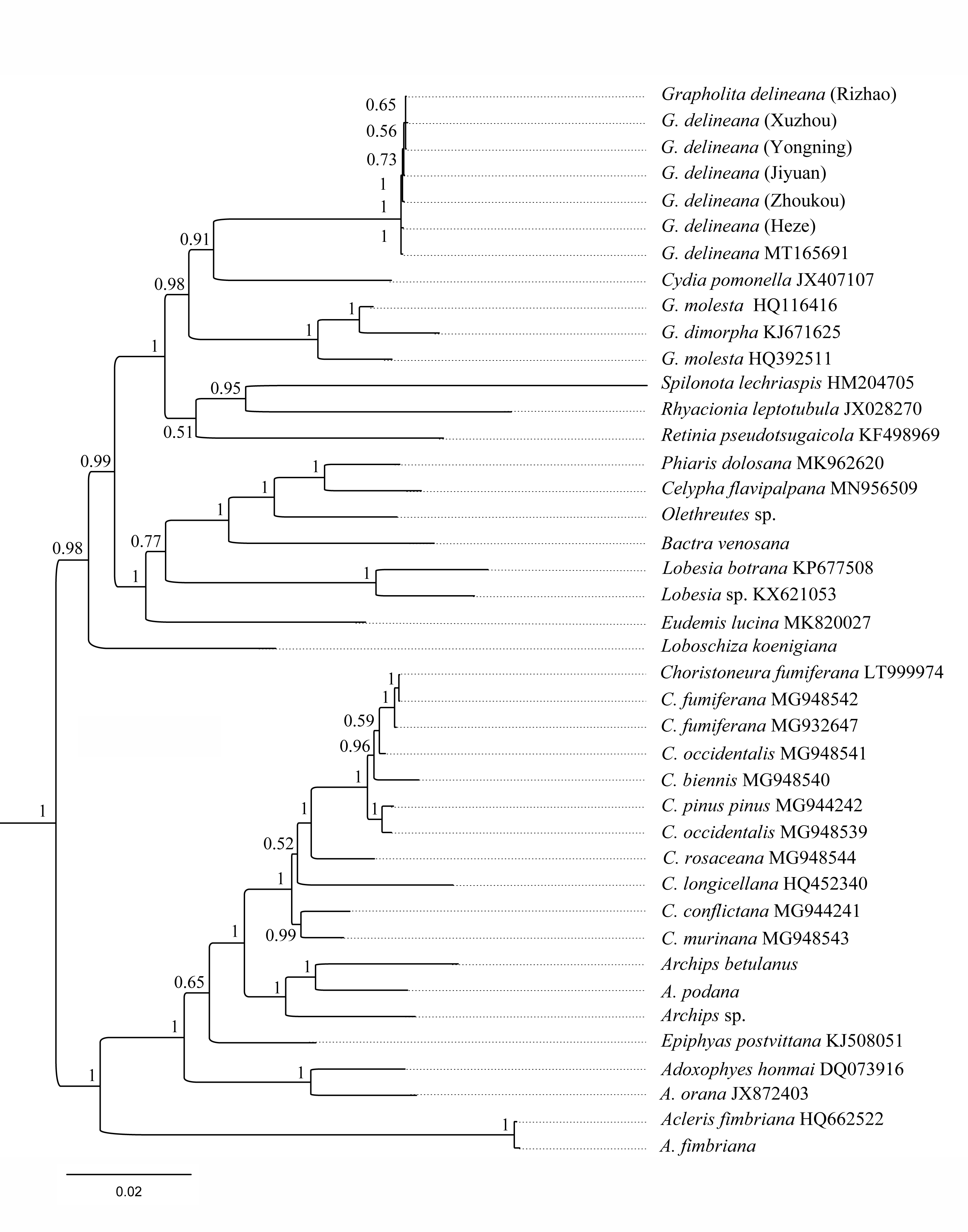

Supplement: Supplementary file 8 — Additional file 8: Figure S3. Phylogenetic tree inferred from Bayesian inference method based on P12 dataset. Numbers on nodes represent the posterior probabilities. [file 12864_2021_8041_MOESM8_ESM.tif]

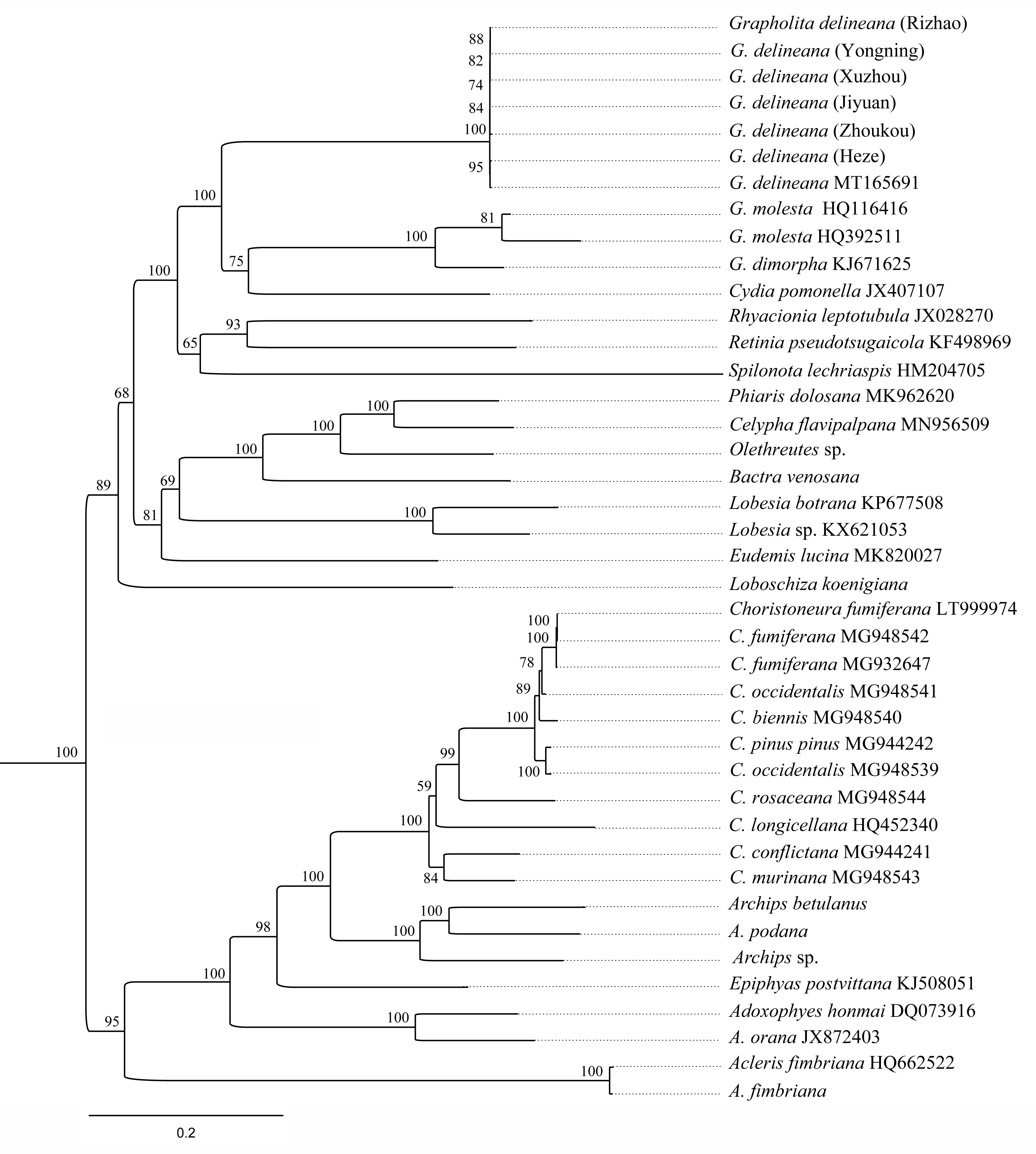

Supplement: Supplementary file 9 — Additional file 9: Figure S4. Phylogenetic tree inferred from maximum likelihood method based on P123 dataset. Numbers on nodes represent the bootstrap supports. [file 12864_2021_8041_MOESM9_ESM.tif]

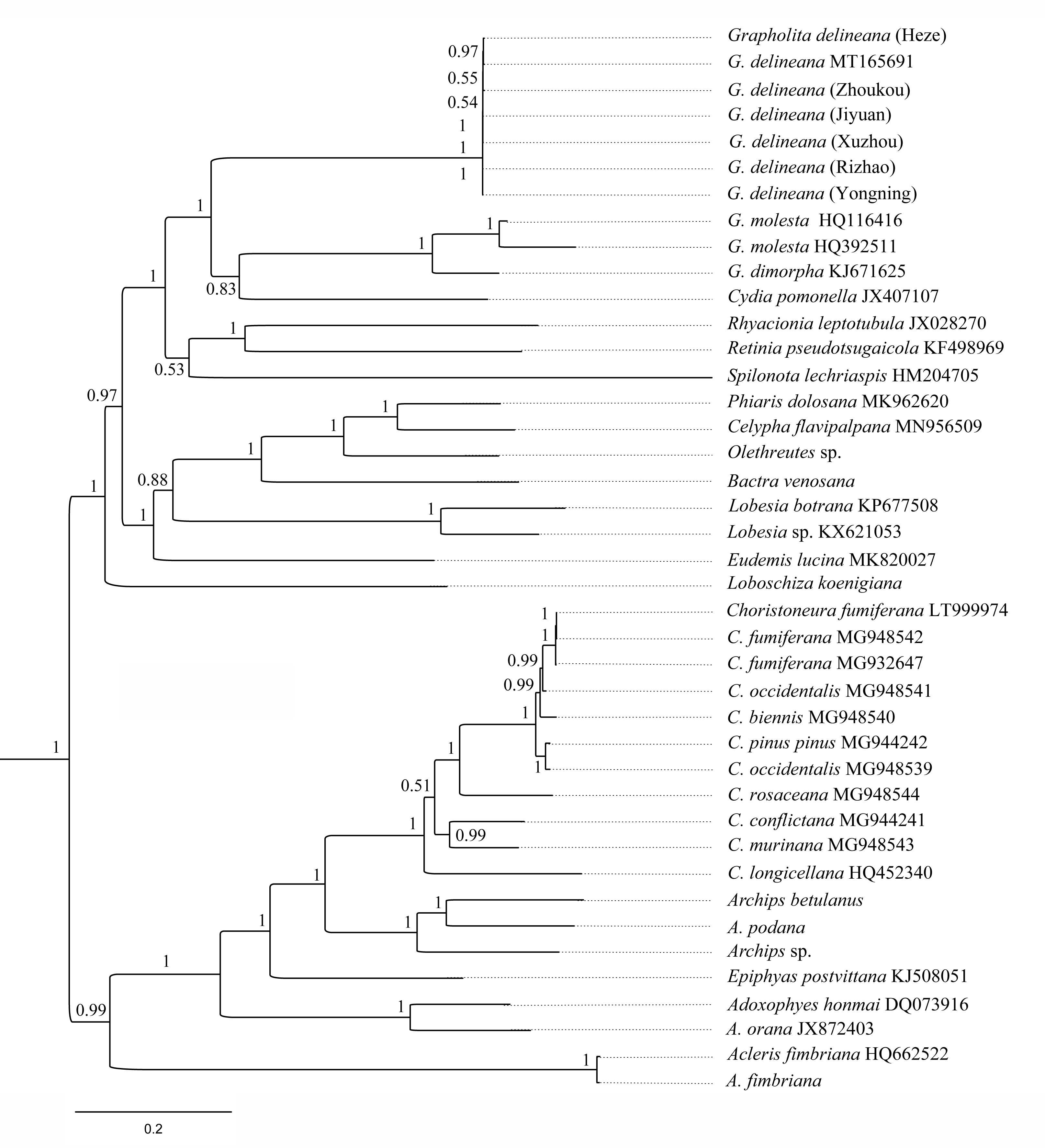

Supplement: Supplementary file 10 — Additional file 10: Figure S5. Phylogenetic tree inferred from Bayesian inference method based on P123 dataset. Numbers on nodes represent the posterior probabilities. [file 12864_2021_8041_MOESM10_ESM.tif]

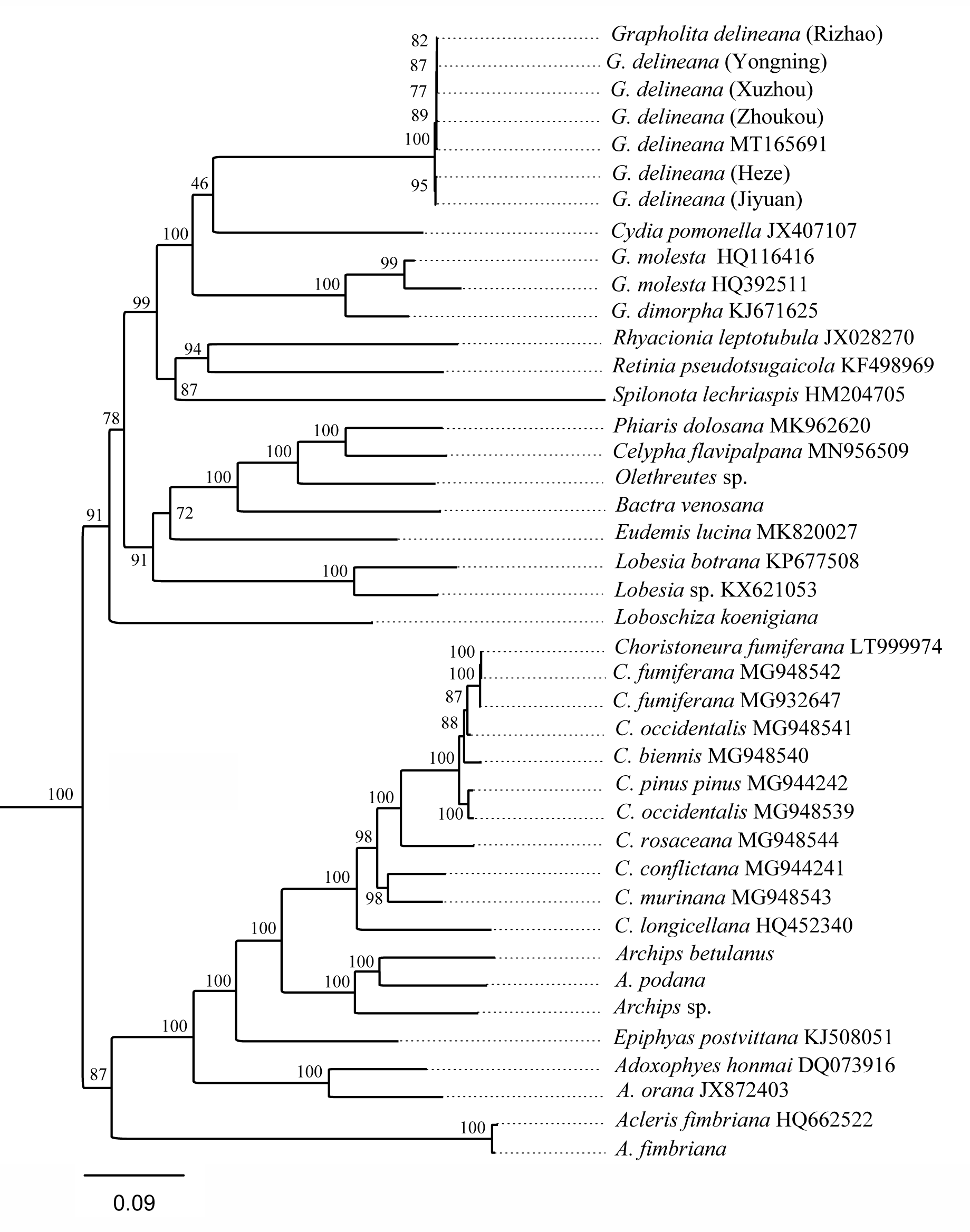

Supplement: Supplementary file 11 — Additional file 11: Figure S6. Phylogenetic tree inferred from maximum likelihood method based on P123R dataset. Numbers on nodes represent the bootstrap supports. [file 12864_2021_8041_MOESM11_ESM.tif]

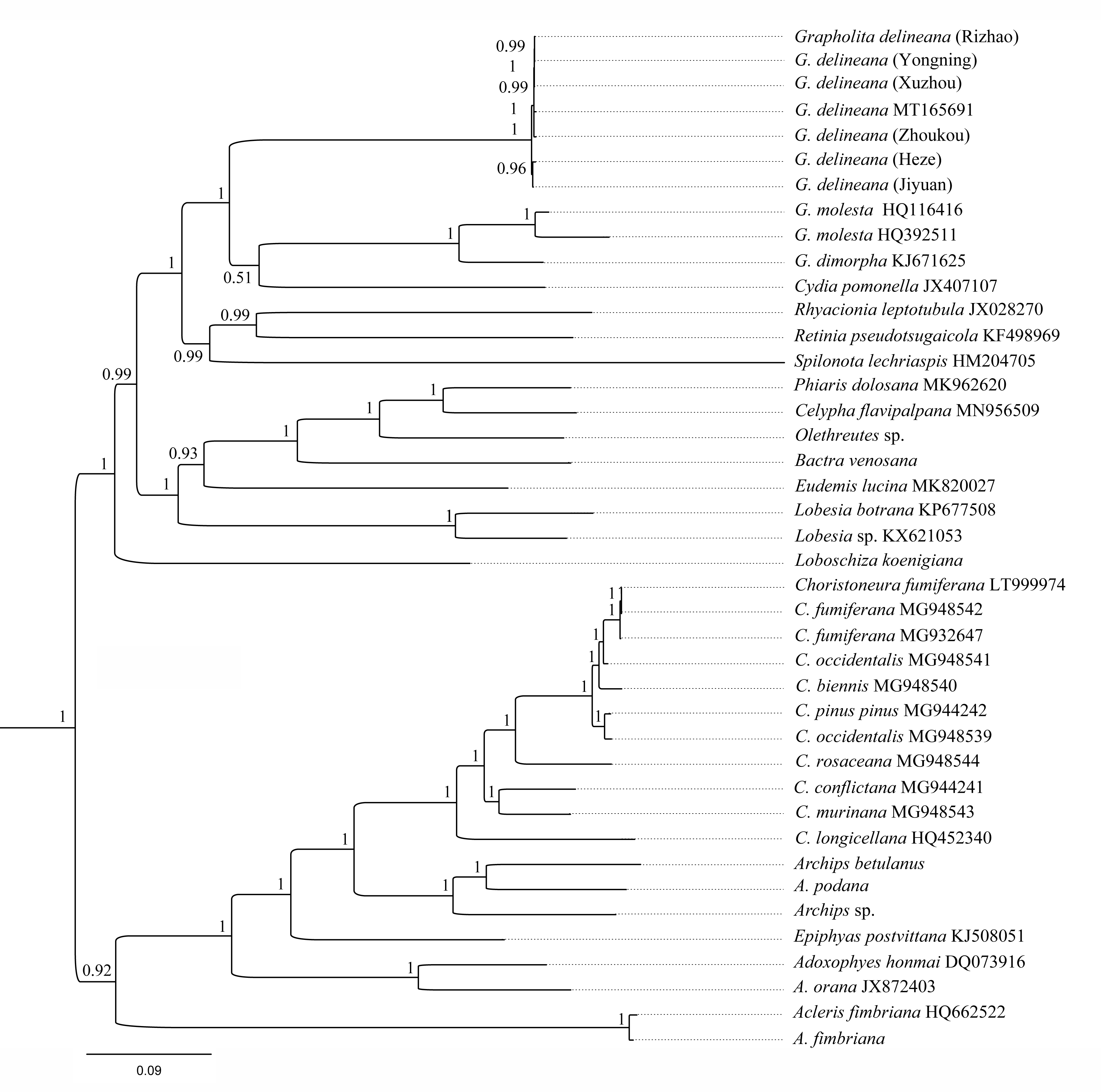

Supplement: Supplementary file 12 — Additional file 12: Figure S7. Phylogenetic tree inferred from Bayesian inference method based on P123R dataset. Numbers on nodes represent the posterior probabilities. [file 12864_2021_8041_MOESM12_ESM.tif]
