# Supplementary material for: The mitochondrial genomes of Tortricidae: nucleotide composition, gene variation and phylogenetic performance
Source: BMC Genomics. 2021 Oct 21;22:755. doi: 10.1186/s12864-021-08041-y (PMC8532297; doi:10.1186/s12864-021-08041-y)
Supplement: Supplementary file 13 — Additional file 13: Table S6. Information of samples sequenced in this study. [file 12864_2021_8041_MOESM13_ESM.docx]

**Table S6. Information of samples with mitogenomes sequenced in this study**

| **Tribe** | **Species/individual** | **Specimen acc.** | **Locality** | **Longitude/ latitude** | **Collection time** |
| --- | --- | --- | --- | --- | --- |
| Enarmoniini | *Loboschiza koenigiana* | 2018GX00012 | Duan, Guangxi, China | 108°05′59″E/23°55′53″N | Sep. 2018 |
| Olethreutini | *Bactra venosana* | 2019HN00054 | Zhoukou, Henan, China | 114°40′44″E/33°38′37″N | Sep. 2019 |
|  | *Olethreutes* sp. | 2019HN00063 | Jigongshan, Henan, China | 114°03′59″E/31°49′14″N | Jul. 2019 |
| Grapholitini | *Grapholita delineana* (Heze) | 2019SD00012 | Heze, Shandong, China | 115°16′00″E/35°29′31″N | Sep. 2019 |
|  | *Grapholita delineana* (Jiyuan) | 2019HN00096 | Jiyuan, Henan, China | 112°23′17″E/35°07′08″N | Jul. 2019 |
|  | *Grapholita delineana* (Zhoukou) | 2019HN00056 | Zhoukou, Henan, China | 114°40′44″E/33°38′37″N | Sep. 2019 |
|  | *Grapholita delineana* (Rizhao) | 2019SD00013 | Rizhao, Shandong, China | 119°31′40″E/35°24′41″N | Sep. 2019 |
|  | *Grapholita delineana* (Xuzhou) | 2019JS00002 | Xuzhou, Jiangsu, China | 117°17′37″E/34°16′09″N | Sep. 2019 |
|  | *Grapholita delineana* (Yongning) | 2019NX00003 | Yongning, Ningxia, China | 106°14′50″E/38°21′14″N | Sep. 2019 |
| Archipini | *Archips podana* | 2019HN00064 | Jigongshan, Henan, China | 114°03′59″E/31°49′14″N | Jul. 2019 |
|  | *Archips betulanus* | 2019HN00065 | Jigongshan, Henan, China | 114°03′59″E/31°49′14″N | Jul. 2019 |
|  | *Archips* sp. | 2019HN00066 | Jigongshan, Henan, China | 114°03′59″E/31°49′14″N | Jul. 2019 |
| Tortricini | *Acleris fimbriana* | 2019HN00055 | Zhoukou, Henan, China | 114°40′44″E/33°38′37″N | Sep. 2019 |
