# Supplementary material for: The mitochondrial genomes of Tortricidae: nucleotide composition, gene variation and phylogenetic performance
Source: BMC Genomics. 2021 Oct 21;22:755. doi: 10.1186/s12864-021-08041-y (PMC8532297; doi:10.1186/s12864-021-08041-y)
Supplement: Supplementary file 14 — Additional file 14: Table S7. The partitioning schemes and corresponding substitution models determined by ModelFinder. [file 12864_2021_8041_MOESM14_ESM.doc]

**Table S7. The partitioning schemes and corresponding substitution models determined by ModelFinder**

| **Partitions** | **Models** | **Genes** |
| --- | --- | --- |
| P1 | TIM2+F+R4 | c1p1, c2p1, c3p1, a6p1, cbp1 |
| P2 | TVM+F+R3 | c1p2, c2p2, c3p2, a6p2, cbp2 |
| P3 | K3Pu+F+R7 | c1p3, c2p3, n2p3, c3p3, n3p3, a6p3, n6p3, a8p3, cbp3 |
| P4 | TPM2+F+R3 | n1p1, n4p1, n5p1, n4lp1 |
| P5 | TIM+F+R3 | n1p2, n4p2, n5p2, n4lp2 |
| P6 | TIM+F+R4 | n1p3, n4p3, n5p3, n4lp3 |
| P7 | TIM2+F+I+G4 | n2p1, n3p1, n6p1, a8p1 |
| P8 | TPM3+F+R3 | n2p2, n3p2, n6p2, a8p2 |
| P9 | GTR+F+I+G4 | *rrnS*, *rrnL* |
| P10 | TVM+F+I+G4 | tRNAs­ |

Note: c1–c3, n1–n6, a6, a8 and cb indicate the 13 PCGs; the p1, p2 and p3 indicate the first, second and third codon positions of each PCG respectively.
