# Supplementary material for: The mitochondrial genomes of Tortricidae: nucleotide composition, gene variation and phylogenetic performance
Source: BMC Genomics. 2021 Oct 21;22:755. doi: 10.1186/s12864-021-08041-y (PMC8532297; doi:10.1186/s12864-021-08041-y)
Supplement: Supplementary file 15 — Additional file 15: Table S8. The partitioning schemes and corresponding substitution models determined by PartitionFinder. [file 12864_2021_8041_MOESM15_ESM.docx]

**Table S8. The partitioning schemes and corresponding substitution models determined by PartitionFinder**

| **Partitions** | **Models** | **Genes** |
| --- | --- | --- |
| P1 | TRN+G | c1p1 |
| P2 | HKY+I | c1p2 |
| P3 | K81UF+I+G | n3p3, a6p3, a8p3, n2p3, n6p3, c1p3, c2p3, c3p3, cbp3 |
| P4 | K81UF+I+G | n4p1, n4lp1, n5p1, n1p1 |
| P5 | GTR+I+G | c3p2, c2p2, n3p2, a6p2, n2p2, cbp2, n4p2, n1p2, n5p2 |
| P6 | TIM+G | n4p3, n1p3, n4lp3, n5p3 |
| P7 | GTR+I+G | a6p1, c2p1, cbp1, c3p1 |
| P8 | GTR+I+G | a8p1, n6p1, n3p1, n2p1 |
| P9 | TVM+G | n4lp2, a8p2, n6p2 |
| P10 | GTR+I+G | *rrnS*, *rrnL* |
| P11 | TVM+I+G | tRNAs |

Note: c1–c3, n1–n6, a6, a8 and cb indicate the 13 PCGs; the p1, p2 and p3 indicate the first, second and third codon positions of each PCG respectively.
